# Effects of 2,4-dichlorophenoxyacetic acid (2,4-d) on the liver and kidney of *Tropidurus torquatus* (Squamata, Tropiduridae)

**DOI:** 10.1007/s10646-026-03170-0

**Published:** 2026-10-23

**Authors:** Letícia Menezes Freitas, Rafael Menezes da Costa, Lucas José Santos Mascarenhas, Leiny Paula de Oliveira, Fabiano Campos Lima, Süleyman Kaplan, Valcinir Aloísio Scalla Vulcani

**Affiliations:** 1 School of Veterinary and Animal Science, Federal University of Goiás, Goiânia, Goiás Brazil; 2 Institute of Health Sciences, Federal University of Jataí, Jataí, Goiás Brazil; 3 Veterinary Medicine Program, Universe University Center Goiânia, Goiânia, Goiás Brazil; 4 Pontifical Catholic University of Goiás, Goiânia, Goiás Brazil; 5 Institute of Biosciences, Federal University of Jataí, Jataí , Goiás Brazil; 6 Department of Histology and Embryology, Ondokuz Mayıs University, Samsun, Samsun Turkey; 7 Institute of Agricultural Sciences, Federal University of Jataí, Jataí , Goiás Brazil

**Keywords:** Pesticide, Oxidative stress, Hepatic, Renal, Reptile

## Abstract

The herbicide 2,4-dichlorophenoxyacetic acid (2,4-D) is widely used in tropical agriculture, yet its effects on non-target ectothermic vertebrates remain poorly understood. This study assessed the subchronic toxicological impact of 2,4-D on the liver and kidneys of *Tropidurus torquatus*, a neotropical lizard commonly found in agricultural areas. Seventeen adult males were divided into control (*n* = 9) and treatment (*n* = 8) groups. The treated group received 0.01 mg/kg body weight/day of 2,4-D orally for 30 days, based on the acceptable daily intake for mammals. Histopathological, stereological, and biochemical analyses were conducted. Hepatic histopathology showed no significant alterations, but stereology revealed a reduction in hepatocyte volume fraction in treated animals (*p* < 0.05). Renal histopathology showed significant degenerative changes, including hydropic-vacuolar degeneration, coagulative necrosis, and accumulation of brownish granular pigment (*p* < 0.0001). Biochemically, 2,4-D exposure triggered oxidative stress in both organs, with increased reactive oxygen species and lipid peroxidation, and reduced activities of superoxide dismutase and catalase. Despite these functional changes, renal stereology showed no significant differences in kidney volume or glomerular count. These findings indicate that even at doses considered safe for mammals, 2,4-D causes subclinical but relevant toxic effects in reptiles. This highlights the limitations of extrapolating mammalian safety thresholds to other taxonomic groups and reinforces the need for specific ecotoxicological assessments focused on tropical herpetofauna.

## Introduction

The herbicide 2,4-dichlorophenoxyacetic acid (2,4-D) is one of the most widely used pesticides worldwide, with extensive application in soybean, corn, and sugarcane crops, especially in tropical countries such as Brazil. According to data from ANVISA, 2,4-D ranks among the most commercially sold pesticides nationally and is frequently detected in environmental samples of soil, water, and sediments from agricultural regions (ANVISA 2023; IBAMA 2026). Despite its regulatory approval based on mammalian toxicity assays, growing evidence indicates that subchronic exposures, even within regulatory limits, can induce biochemical, physiological, and cellular alterations in non-model organisms such as fish, amphibians, and reptiles (Weir et al. 2010; Meftaul et al. 2020).

Terrestrial reptile species, particularly tropical lizards, are frequently exposed to agricultural contaminants due to their close contact with the soil, diurnal habits, and wide distribution in anthropized areas. However, these vertebrates remain excluded from standardized toxicological testing, which leads to data extrapolation for species with greatly different physiology and metabolism (Weir et al. 2010; USEPA 2016). As ectotherms, reptiles exhibit temperature-dependent metabolism and low renal and hepatic clearance rates, which may prolong the half-life of xenobiotics such as 2,4-D and potentiate their deleterious effects (Moeller et al. 2013; Caetano and Colli 2021).

From a toxicological perspective, the liver and kidneys are primary target organs during herbicide exposure (Yu and Anderson 1997; Chen et al. 2019). In the liver, 2,4-D may trigger oxidative stress, leading to the production of reactive oxygen species (ROS) and the inhibition of antioxidant enzymes such as superoxide dismutase (SOD) and catalase (CAT). This imbalance compromises membrane and organelle integrity, promoting lipid peroxidation, mitochondrial dysfunction, and ultimately, cell death (Troudi et al. 2012; Matyishyn et al. 2014; Menezes et al. 2015). In the kidney, the main excretion pathway for 2,4-D, it may result in damage to the tubular epithelium, manifested as degeneration and necrosis, thereby impairing renal function and reducing filtration and reabsorption capacity (Troudi et al. 2011; Tayeb et al. 2012; Matyishyn et al. 2014; Menezes et al. 2015).

Despite these risks, studies evaluating the effects of 2,4-D in reptiles using integrated approaches remain scarce. *Tropidurus torquatus*, a lizard widely distributed in the Brazilian Cerrado, represents an ideal bioindicator species due to its abundance, occurrence in agricultural environments and its ecological relevance. Previous studies have demonstrated its sensitivity to histological and biochemical changes in response to environmental stressors, but there is a lack of evidence regarding the toxicity of 2,4-D at doses based on its Acceptable Daily Intake (ADI) (Freitas et al. 2020; Ferreira 2021; ANVISA 2023).

This study aimed to evaluate the effects of subchronic exposure to 2,4-D at a dose of 0.01 mg/kg b.w. on the liver and kidneys of *T. torquatus*, through integrated histopathological, stereological, and biochemical analyses, in order to assess the adequacy of this dose for ectothermic organisms in agriculturally impacted environments.

## Materials and methods

### Ethical approval

Specimen collection was authorized by SISBIO (79530-3), and all procedures were approved by the Animal Ethics Committee of the Federal University of Jataí (CEUA UFJ − 014/2022).

### Lizard collection and maintenance

Seventeen adult male *T. torquatus* (Squamata, Tropiduridae) were captured with handmade nooses in the urban area of Jataí (GO) during the reproductive season (October to February), to avoid potential confounding effects of vitellogenesis-related hepatic changes in females (Gavaud 1986; Niksirat et al. 2025). After capture, the animals were individually transported in cotton bags to the Human and Comparative Anatomy Laboratory at the Federal University of Jataí, where they underwent a 7-day acclimation period (Py-Daniel et al. 2017).

Lizards were randomly assigned to control (CT, *n* = 9) and treatment (TR, *n* = 8) groups and housed in glass terrariums (60 × 50 × 40 cm) with moistened vermiculite substrate, clay tile enrichment, and continuous access to water. Lighting was provided by 50 W UVA/UVB 3.0 lamps, operated on alternating 2-hour on/off cycles between 7 a.m. and 7 p.m., simulating intermittent sunlight exposure. The diet consisted of cockroaches and mealworm larvae provided *ad libitum* (Py-Daniel et al. 2017).

### 2,4-D exposure and euthanasia

Animals in the TR group were exposed to 2,4-D, using the commercial formulation Mirant^®^ (288 g/L, dimethylamine salt), diluted in distilled water and administered by oral gavage at a dose of 0.01 mg/kg body weight/day for 30 consecutive days. This dose was based on the ADI established by the Brazilian Health Regulatory Agency (ANVISA 2025). The CT group received only distilled water, using the same route and volume.

Animals were weighed weekly for individual dose adjustment (Amaral et al. 2012). At the end of the experimental period, all individuals were euthanized via intracardiac administration of propofol (Provive^®^ 1%, 30 mg/kg) by a veterinarian. Propofol was used as a single-step procedure, as its rapid anesthetic action induces immediate deep anesthesia and loss of consciousness, and therefore no additional anesthetic was applied prior to euthanasia. Death was confirmed by the absence of heartbeat, spinal reflexes, and respiratory movements, following Brazilian guidelines for the euthanasia of wild animals (CONCEA 2018; Sladky et al. 2023).

After euthanasia, liver and kidneys were collected and processed for subsequent analyses. The liver was sectioned into fragments approximately 2 mm thick using a 3D-printed slicer designed to standardize sample volume and distribution (Tyson et al. 2015). Hepatic fragments were numbered, divided into two sets (even and odd), and randomly allocated for histological or biochemical analyses. Fragments for biochemical assays were immediately frozen and stored at − 80 °C, while those for histology were fixed in 10% buffered formalin and stored in 70% ethanol.

The right and left kidneys from each animal were also randomly assigned for freezing or fixation, ensuring cross-representativeness between histopathological and biochemical analyses.

### Histopathological analysis

Liver and kidney samples from all animals (CT = 9; TR = 8) were fixed in 10% buffered formalin for 48 h, processed in an automatic tissue processor (HistoCore Arcadia H^®^, Leica Biosystems), embedded in paraffin, and sectioned at 4 μm using a rotary microtome. Sections were stained with hematoxylin and eosin (H&E) in an automated staining system (Autostainer XL^®^, Leica Biosystems).

Slides were examined under a light microscope (DM48^®^, Leica Microsystems) equipped with a DFC700 T^®^ digital camera using a 40× objective (total magnification of 400×) for the liver and a 20x objective (total magnification of 200x) for the kidney. For each organ, six microscopic fields per animal were selected at 400× magnification, within viable and morphologically preserved areas, distributed systematically and equidistantly across the slide, avoiding regions with artifacts, folds, or tissue discontinuity (Howard and Reed 2005).

Given that *T. torquatus* does not exhibit the functional lobular zonation typical of mammalian livers, a selection adapted to the homogeneous architecture of the hepatic parenchyma in reptiles was carried out (Miller and Zachary 2022). Evaluated parameters included melanomacrophage centers, hepatocellular vacuolation, inflammatory infiltrate, fibrosis, necrosis, and inflammation in fibrous tissue. The first three variables were assessed semi-quantitatively using an ordinal intensity scale (0 = absent; 1 = mild; 2 = moderate; 3 = severe) (Table 1). The remaining variables were recorded as presence or absence (Anater et al. 2020; Barboza et al. 2023).

**Table 1:** Scoring criteria used for histopathological evaluation of the liver in *Tropidurus torquatus*, adapted from Anater et al. (2020) and Barboza et al. (2023)

	Melanomacrophages	Hepatic vacuolization	Inflammatory infiltrate
Mild (1)	Up to 30% of the field, clustered or dispersed	Up to 30% of the field, clear cytoplasmic area	Up to 30% of the field, dispersed mononuclear cells, no granulomas
Moderate (2)	30–70% of the field, clustered or dispersed	30–70% of the field, clear cytoplasmic area	30–70% of the field, dispersed mononuclear cells, no granulomas
Severe (3)	Over 70% of the field, clustered or dispersed	Over 70% of the field, clear cytoplasmic area	Over 70% of the field, dispersed mononuclear cells, no granulomas

For the kidneys, given the lack of a clear distinction between cortex and medulla in *T. torquatus*, the fields were selected based on the presence of glomeruli and proximal and distal convoluted tubules. The presence or absence of necrosis, brownish granular pigment, glomerular lesions, tubular alterations, interstitial inflammatory infiltrate, and vascular congestion were evaluated, with only the first two being found (Soares and Fava-de-Moraes 1984; Brunk and Terman 2002; Rheubert et al. 2011; Viriato et al. 2021).

All analyses were conducted by a veterinary pathologist blinded to group identity, following best practices for histological evaluation in toxicological studies involving non-model vertebrates (Howard and Reed 2005).

### Stereological analysis

Stereological analyses were conducted at the LabCell (Federal University of Goiás), LabFish, and Laboratory of Morphophysiology (Federal University of Jataí), using a light microscope and a stereomicroscope equipped with image capture systems. A systematic random sampling method was adopted, and quantitative stereology was performed using Stepanizer v.2b27 software.

For kidneys, total renal volume was estimated using Cavalieri’s principle, with serial equidistant sections and point grid overlays. To estimate total glomerular number, the physical disector probe was employed, enabling unbiased three-dimensional counting between paired sections (Warille et al. 2023).

In the liver, the volume fraction occupied by hepatocytes was estimated using the area fraction fractionator probe, applying systematic random sampling and point-by-point analysis of digitized images. Measurements were performed on H&E-stained histological sections with sufficient resolution for cellular identification. Data were expressed as the relative volumetric proportion of hepatocytes in relation to the total sampled liver volume.

### Biochemical analysis

Liver and kidney samples (*n* = 17) were stored at − 80 °C until analysis, conducted in collaboration with the Pharmacology Laboratory at the Federal University of Jataí and the Cardiovascular Pharmacology Laboratory at the University of São Paulo. Tissues were homogenized in 50 mM phosphate buffer (pH 7.0) using a tube shaker on ice and then centrifuged at 15,000 g for 15 min at 4 °C. The supernatant was collected for biochemical assays.

Intracellular ROS generation was quantified by fluorometry using two fluorescent probes: 2’,7’-dichlorodihydrofluorescein diacetate (H₂DCFDA), which detects non-specific ROS, and dihydroethidium (DHE), used for the specific detection of superoxide anion (O₂•–) (Krishnamurthy et al. 2024).

Lipid peroxidation was assessed by the thiobarbituric acid reactive substances (TBARS) assay, with quantification of malondialdehyde (MDA), one of the main by-products of membrane lipid oxidation (Ohkawa et al. 1979; Krishnamurthy et al. 2024).

The activity of the antioxidant enzymes CAT and SOD was determined by spectrophotometry using commercial kits (Sigma-Aldrich^®^), following the manufacturers’ instructions. All assays were performed in duplicate, and results were expressed as enzymatic activity units per milligram of total protein (Krishnamurthy et al. 2024).

### Statistical analysis

Statistical analyses were performed using BioEstat^®^ v.5.0 software. Data normality was verified using the Lilliefors and Kolmogorov-Smirnov tests. For semiquantitative histopathological scores, the Mann-Whitney test was applied. Qualitative variables (presence/absence) were analyzed using the chi-square (χ²) test. Biochemical variables and renal stereological parameters, considered continuous and normally distributed, were analyzed using Student’s t-test for independent samples. Hepatic stereological data, which did not show normal distribution, were analyzed using the Kruskal-Wallis test followed by Dunn’s post hoc test when applicable. All analyses were performed at a 5% significance level (*p* < 0.05).

## Results

### Histopathological analysis

**Fig. 1:**
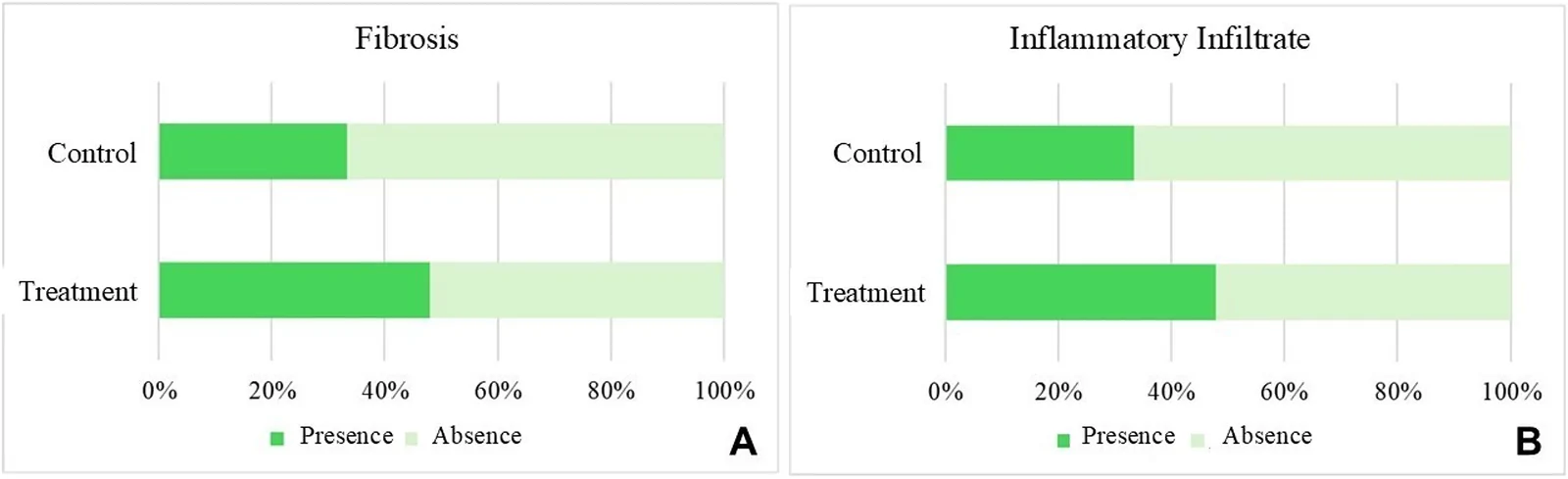
Relative frequency of presence and absence of **A** fibrosis and **B** inflammatory infiltrate in the liver of *Tropidurus torquatus*. No significant difference was observed between groups (*p* > 0.05)

In the liver sections of *T. torquatus*, inflammation associated with fibrosis was observed as scattered mononuclear cell accumulation within the connective tissue, along with collagen fiber deposition. Fibrosis was classified as mild in most individuals, and hepatocellular necrosis was not identified in any of the evaluated fields. There was no statistically significant difference between the CT and TR groups regarding the occurrence of fibrosis or inflammation (*p* > 0.05) (Fig. 1).

**Fig. 2:**
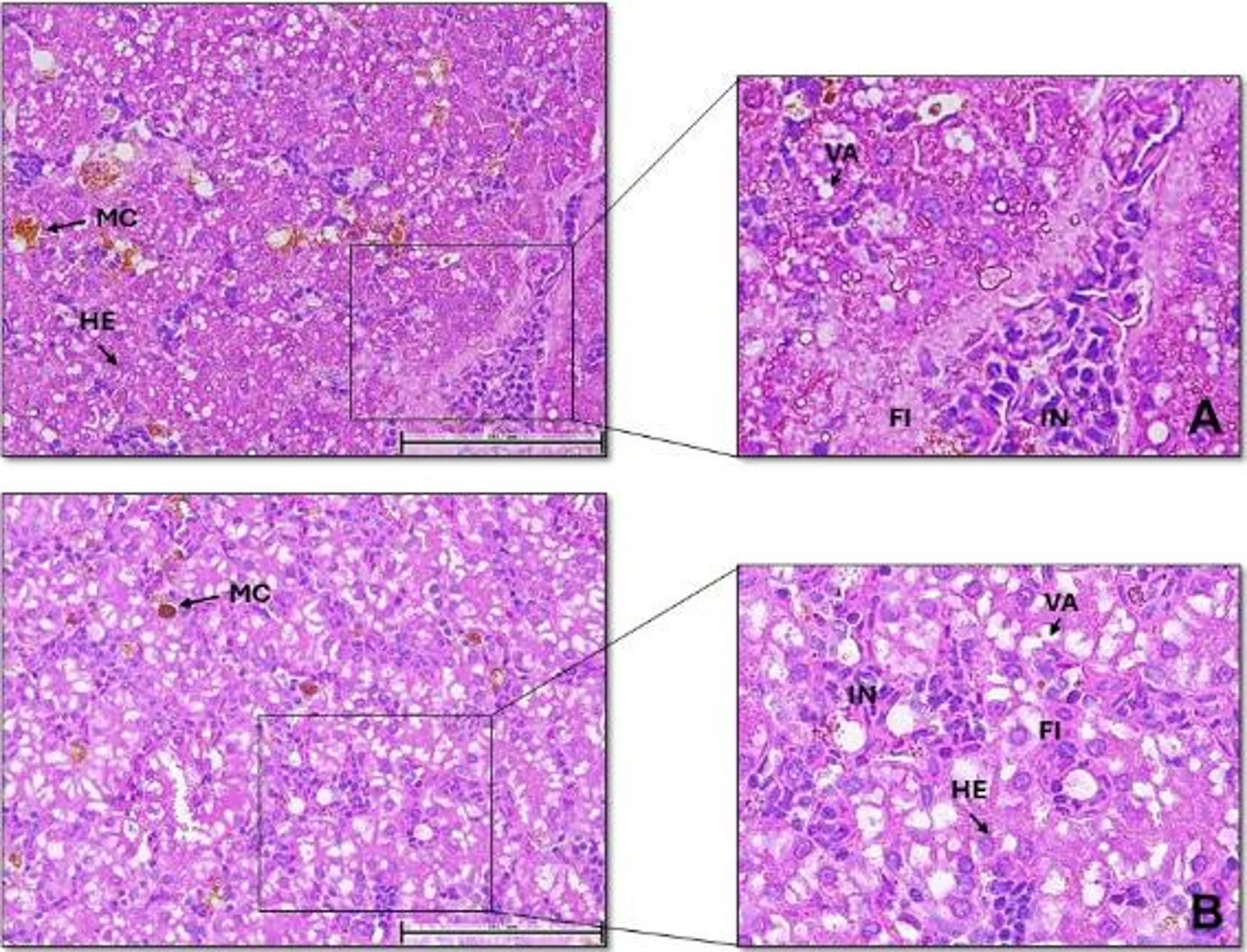
Representative photomicrographs of the liver of *Tropidurus torquatus.***A** Control group (CT). **B** Treatment group (TR). Severe vacuolization (VA) in hepatocytes (HE), presence of melanomacrophage centers (MC), mild fibrosis (FI), and mononuclear infiltrate (IN) are observed. H&E staining, 40× magnification. Scale: 50 μm

In the liver parenchyma, cytoplasmic vacuolization of varying degrees was observed in hepatocytes, visualized as clear intracytoplasmic spaces with well-defined borders. Vacuolization was classified as pronounced in most of the evaluated fields in both groups. Melanomacrophage centers were identified as clusters of pigmented cells with brown to black coloration, distributed mainly in the perivascular region. Statistical analysis of the semi-quantitative scores revealed no difference between the CT and TR groups regarding the intensity of vacuolization and the number of melanomacrophage centers, suggesting that these parameters are naturally present in the species and are not due to the herbicide (Fig. 2; Table 2).

**Table 2:** Median histopathological scores for liver tissue (0 = absent, 1 = mild, 2 = moderate, 3 = severe) in control (CT) and treated (TR) groups of *Tropidurus torquatus*

Groups	Melanomacrophages	Hepatic vacuolization	Inflammatory infiltrate
Control (CT)	1	3	1
Treatment (TR)	1	3	1

**Fig. 3:**
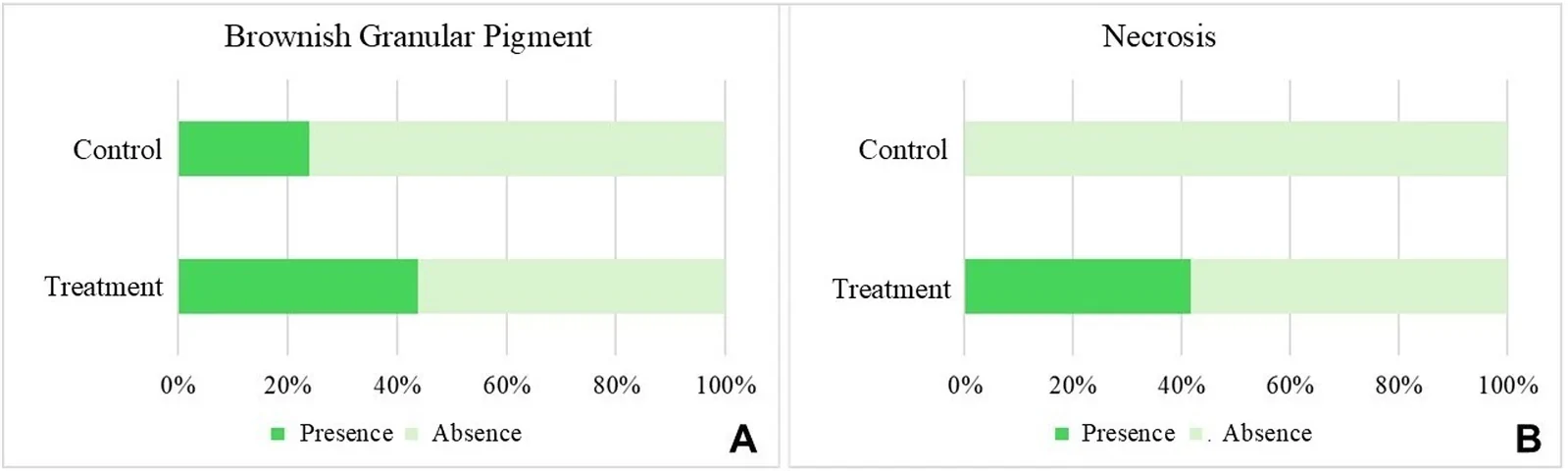
Relative frequency of presence and absence of **A** brownish granular pigment and **B** necrosis in the kidneys of *Tropidurus torquatus*. There was a statistically significant difference between the groups for both parameters (A: *p* = 0.035; B: *p* < 0.0001)

**Fig. 4:**
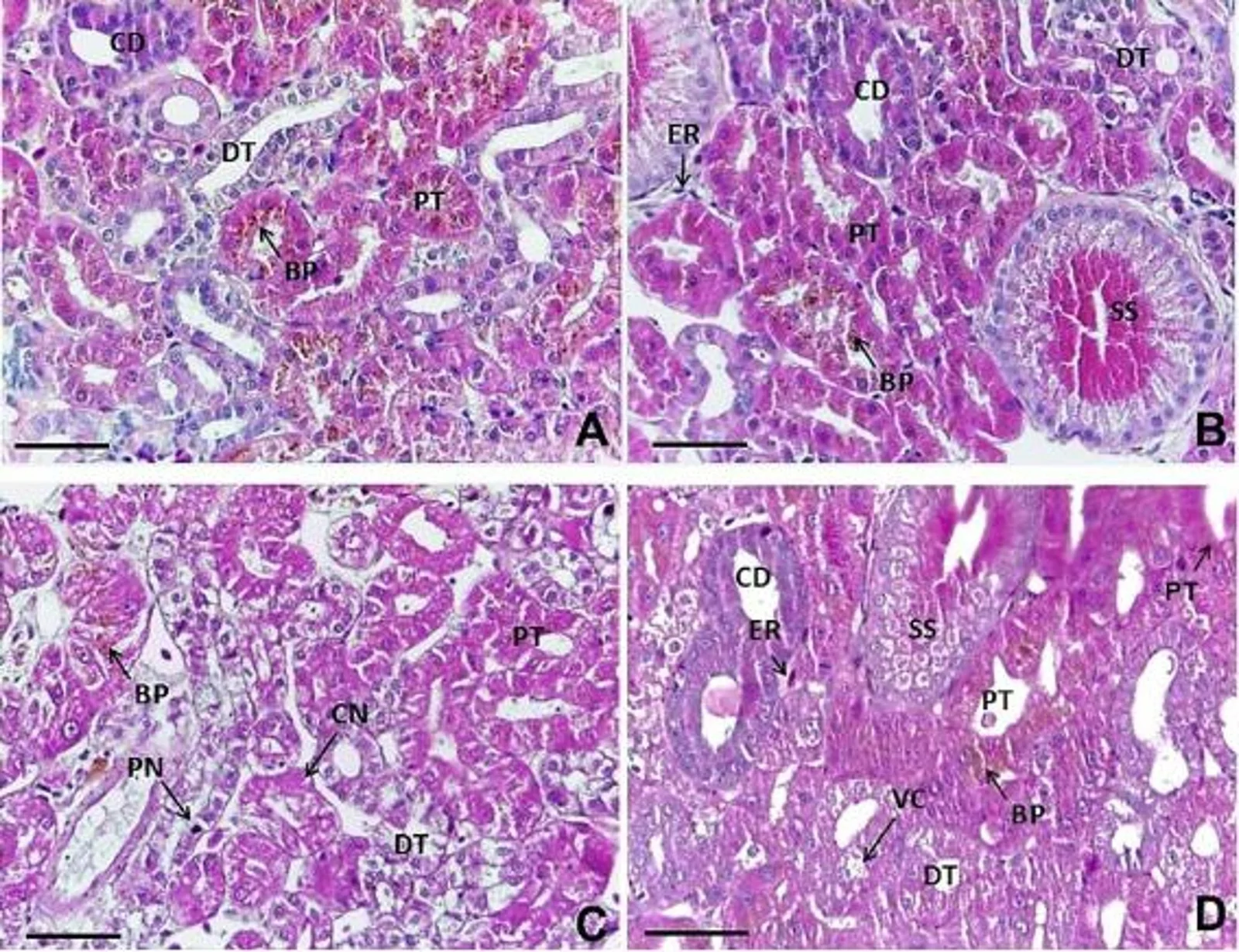
Representative histological photomicrographs of *Tropidurus torquatus* kidney sections. **A** and **B** – Control (CT). **C** and **D** – Treatment (TR). Renal structures such as proximal tubules (PT), distal tubules (DT), collecting ducts (CD), and sexual segment (SS), as well as erythrocytes (ER) in the capillaries, are visible. Mild to moderate amounts of intracellular brown pigment (BP) are observed. In **C** and **D**, renal tubules display diffuse, moderate to severe hydropic-vacuolar degeneration, characterized by clear cytoplasmic vacuolization (VC) and increased cell volume. Multiple areas of coagulative tubular necrosis (CN) with eosinophilic cytoplasm, preserved cell contours, and pyknotic (PN), karyorrhectic, or absent (karyolytic) nuclei are also evident, indicating cell death. H&E staining, 40× magnification. Scale: 50 μm

In the kidneys of *T. torquatus*, no glomerular alterations, interstitial inflammatory infiltrate, or vascular congestion were observed in either group. The presence of brown granular pigment in the tubular epithelium was detected in both groups, with a higher frequency in the TR group compared to CT (*p* = 0.035). Furthermore, the presence of tubular necrosis was significantly associated with the experimental group, being absent in the control group (*p* < 0.0001; Figs. 3 and 4).

### Stereological analysis

**Fig. 5:**
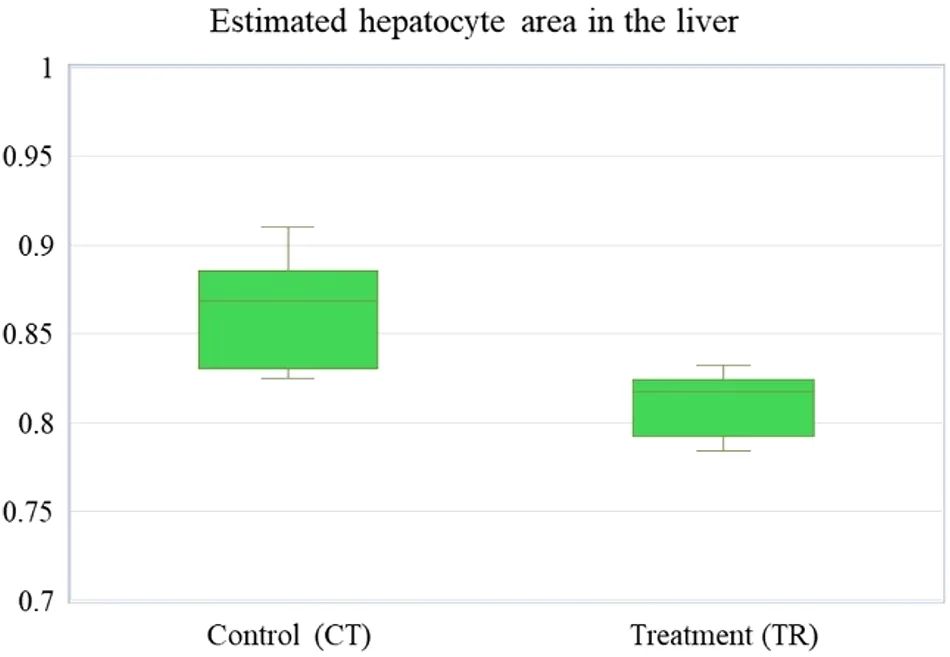
Stereological estimation of the volume fraction occupied by hepatocytes in the liver of *Tropidurus torquatus* in the control (CT) and 2,4-D treated (TR) groups. The analysis was performed using an area fraction fractionator probe on histological sections stained with hematoxylin and eosin (H.E.). Each point represents the individual mean of six fields per animal (CT, *n* = 9; TR, *n* = 8). In the boxplot, the box delimits the interquartile range (IQR), between the first (Q1) and third quartiles (Q3), the inner line corresponds to the median (Q2), and the whiskers represent the minimum and maximum values within 1.5 times the IQR. A statistically significant difference was observed between the groups (Student’s t-test; *p* < 0.05)

In the liver of *T. torquatus*, the volumetric fraction occupied by hepatocytes was estimated using stereology with the area fraction fractionator probe. Animals from the CT group showed an average of 85.0% of liver volume occupied by hepatocytes, whereas in the TR group, this mean was 81.0%. The difference between groups was statistically significant (*p* < 0.05) (Fig. 5).

**Fig. 6:**
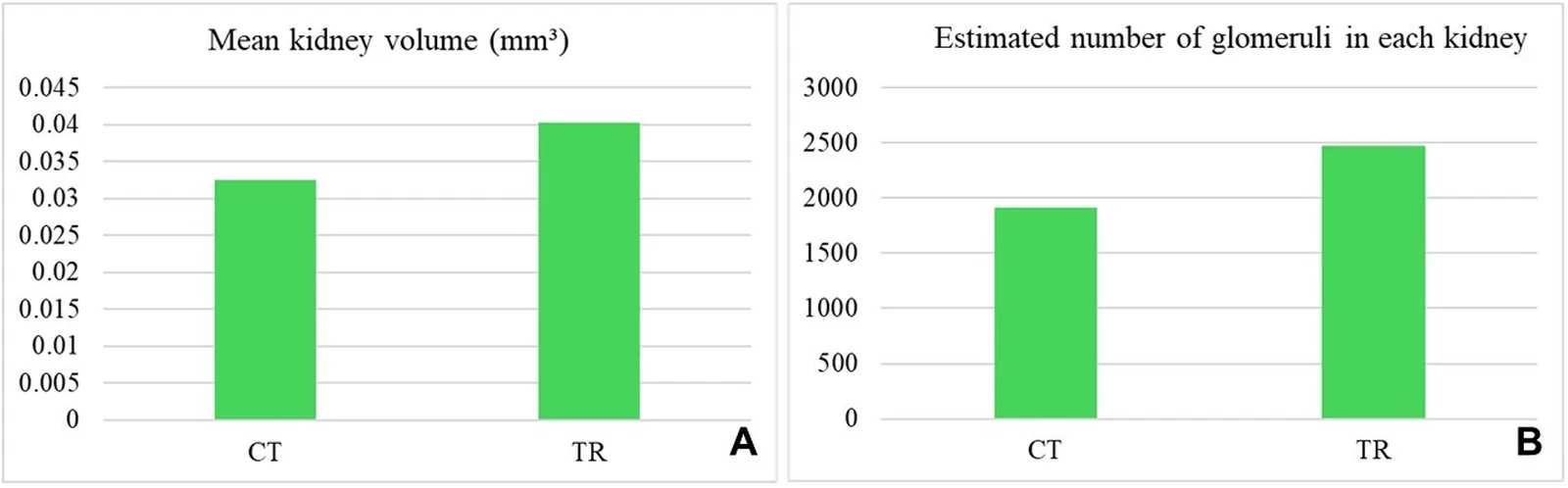
Stereological parameters of the kidneys of *Tropidurus torquatus* in control (CT) and 2,4-D treated (TR) groups. **A** Estimated renal volume (in mm³) using Cavalieri’s principle. **B** Total estimated number of glomeruli per kidney, obtained with the physical disector probe. Analyses were conducted on histological sections of one kidney (right or left), randomly allocated for each animal (CT, *n* = 9; TR, *n* = 8). No statistically significant differences were found between groups (Student’s t-test; *p* > 0.05)

In the kidneys, total renal volume was estimated based on Cavalieri’s principle, and total glomerular number was assessed using the physical disector probe. Analyses were performed on one kidney (right or left) per animal, randomly allocated. No statistically significant differences were observed between groups for mean renal volume (*p* > 0.05) or total estimated number of glomeruli per kidney (*p* > 0.05) (Fig. 6).

### Biochemical analysis

**Fig. 7:**
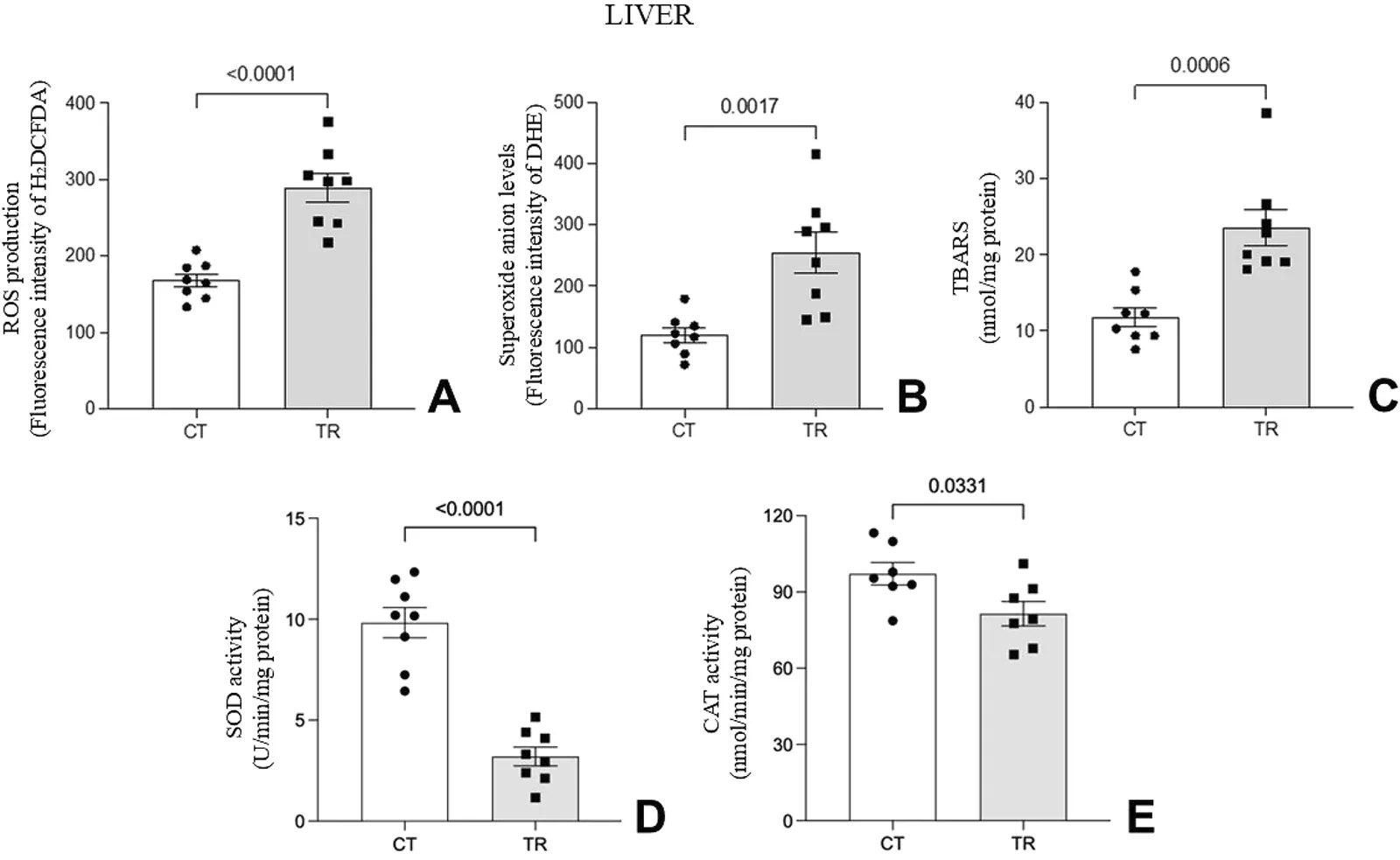
Biochemical markers of oxidative stress in the liver of *Tropidurus torquatus*. **A** Production of nonspecific ROS (H₂DCFDA probe; relative fluorescence, UA). **B** Detection of superoxide anion (DHE probe; relative fluorescence, UA). **C** Malondialdehyde (MDA; nmol MDA mg⁻¹ protein) levels by TBARS assay. **D** Superoxide dismutase (SOD) activity; U mg⁻¹ protein). **E** Catalase (CAT) activity; µmol H₂O₂ min⁻¹ mg⁻¹ protein). Control group (CT, *n* = 9); 2,4-D treated group (TR, *n* = 8). Each point represents the individual value for an animal, and the bars indicate mean ± standard error of the mean (SEM). The values above the bars correspond to the significance level (p) obtained by the Student’s t-test

Exposure to 2,4-D resulted in significant alterations in oxidative stress markers in the liver and kidney of *T. torquatus*. In the liver, there was a significant increase in the production of ROS, both nonspecific (detected by H₂DCFDA) and superoxide-specific (detected by DHE), in the TR group compared to CT. Additionally, malondialdehyde (MDA) levels, indicative of lipid peroxidation through the TBARS assay, were also significantly higher in the TR group. Regarding antioxidant enzymes, SOD activity was significantly reduced in the liver of treated animals. CAT activity was also lower in the TR group, with a statistically significant difference (Fig. 7).

**Fig. 8:**
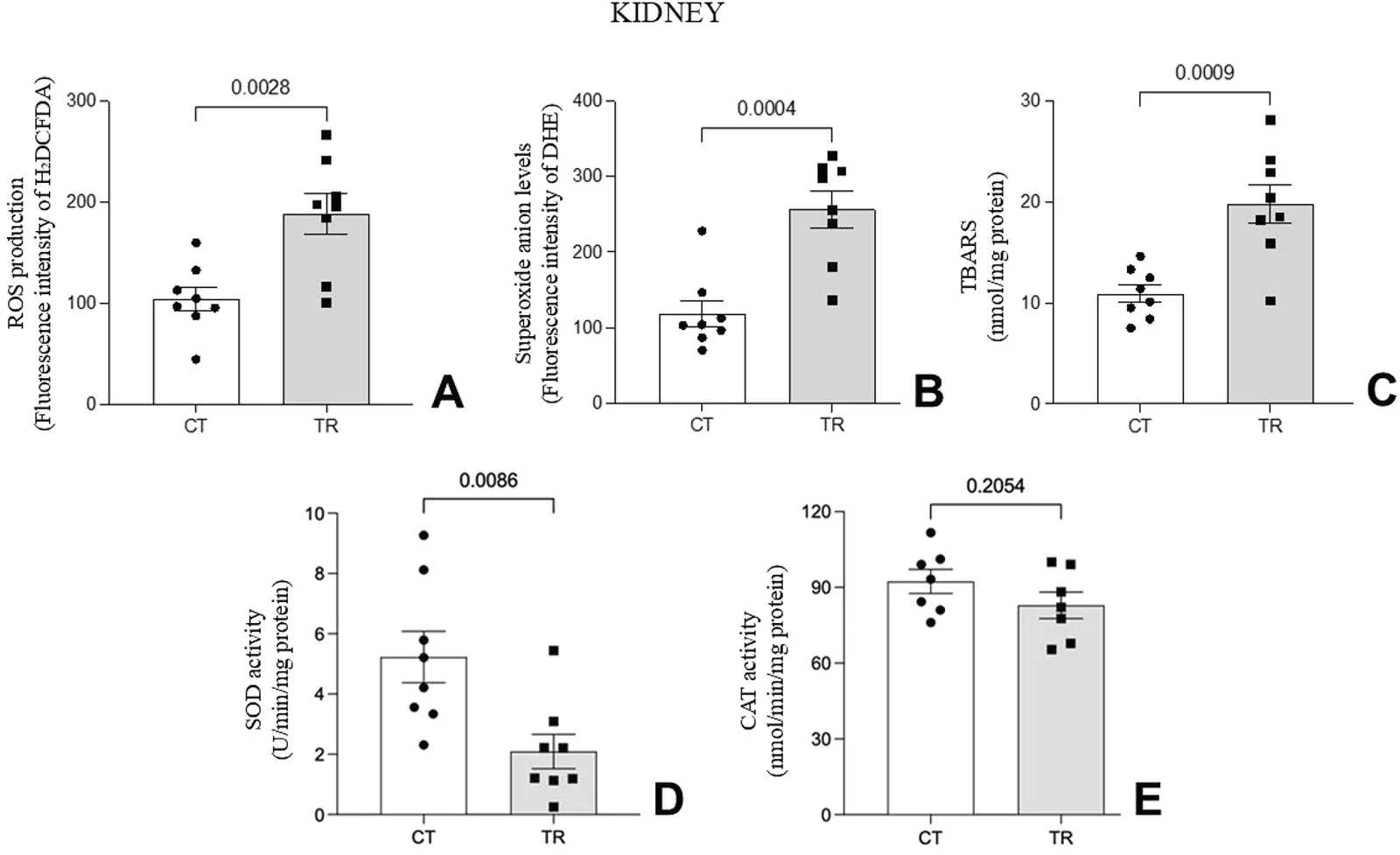
(A) Production of nonspecific ROS (H₂DCFDA probe; relative fluorescence, UA). **B** Detection of superoxide anion (DHE probe; relative fluorescence, UA). **C** Malondialdehyde (MDA; nmol MDA mg⁻¹ protein) levels by TBARS assay. **D** Superoxide dismutase (SOD; U mg⁻¹ protein) activity. **E** Catalase (CAT; µmol H₂O₂ min⁻¹ mg⁻¹ protein) activity. Control group (CT, *n* = 9); 2,4-D treated group (TR, *n* = 8). Each point represents the individual value for an animal, and the bars indicate mean ± standard error of the mean (SEM). The values above the bars correspond to the significance level (p) obtained by the Student’s t-test

In the kidneys, the TR group showed a significant increase in both nonspecific ROS and superoxide anion compared to CT. MDA levels were also significantly elevated. SOD activity was significantly lower in the treated group. Although CAT activity was reduced in the TR group, it did not present a statistically significant difference (Fig. 8).

## Discussion

The results obtained in this study reinforce the importance of directly investigating the effects of agrochemicals on reptiles, such as *T. torquatus*, since these ectothermic organisms exhibit metabolic pathways, detoxification mechanisms, and renal physiology that differ from the mammals traditionally used in toxicological assays (Tayeb et al. 2011; Holz 2020; Fasola et al. 2022). Subchronic exposure to 2,4-D, even at a dose equivalent to the ADI for mammals (0.01 mg/kg b.w.), was capable of inducing significant biochemical alterations and focal histopathological lesions in the liver and kidney of *T. torquatus*, revealing early pathophysiological effects even in the absence of diffuse morphometric alterations detectable by conventional stereology, indicating a pattern of early functional toxicity in understudied ectothermic organisms (Bonfim et al. 2020; Martins et al. 2024).

Given the widespread use of 2,4-D in agricultural areas and its proven capacity to reach adjacent ecosystems via atmospheric drift, surface runoff, or leaching, the findings of this study gain extended ecological relevance, especially considering the vulnerability of non-target organisms such as reptiles (Rice et al. 2010; Huertas et al. 2015). Chronic, repeated, or cumulative exposure to this herbicide may compromise key physiological functions, such as detoxification mechanisms, energy metabolism, and cell membrane integrity, directly affecting critical parameters such as longevity, fecundity, and survival rates of exposed species.

In a study comparable to the present one, Bonfim et al. (2020) exposed rats to 2,4-D at doses consistent with manufacturer recommendations. Although no hepatic necrosis or cholestasis was observed, mild steatosis and inflammation were reported, along with discrete renal inflammation and hydropic degeneration of tubular epithelial cells. These findings reinforce that even exposure levels considered safe may induce subtle biochemical and histological alterations, as also evidenced in *T. torquatus* in the present study.

In the liver, no severe morphological alterations such as extensive necrosis or diffuse fibrosis were detected. The presence of hepatocellular vacuolization and melanomacrophage centers in both groups is consistent with natural histological features of the species (Firmiano et al. 2011). However, hepatic stereology revealed a significant reduction in the volume fraction occupied by hepatocytes in treated animals, suggesting an adaptive or functional response to the xenobiotic. This response may be associated with metabolic alterations induced by oxidative stress and cellular reorganization, as described for subchronic exposure to environmental contaminants (Mahalingaiah et al. 2017; Hsu and Tain 2021).

This reduction can be interpreted in light of the mobilization of energy reserves, such as lipids and glycogen, under conditions of increased metabolic demand and oxidative stress. In reptiles, hepatic cytoplasmic vacuoles are commonly associated with these reserves and vary according to energetic balance and physiological state (Schaffner 1998; Firmiano et al. 2011; Odokuma and Omokaro 2015; Nafady and Awadalla 2019; Verderame and Scudiero 2019). The reduction of these structures observed in *T. torquatus* may therefore reflect glycogen mobilization or lipid degradation, consistent with patterns reported following sublethal pesticide exposure (Amaral et al. 2012; Mali et al. 2013; Price et al. 2017; Curi et al. 2019).

Moreover, the degradation of cell membranes induced by radicals such as ROO•, O2-•, and OH• may compromise the structural integrity of organelles, favoring the formation of cytoplasmic vacuoles that reflect not only the mobilization of energy reserves but also the impact of oxidative damage on intracellular membranes. This results in alterations that often escape detection by conventional histopathological analysis (Shubin et al. 2016).

Additionally, biochemical findings confirmed the presence of pronounced oxidative stress in the liver of animals exposed to 2,4-D, with increased ROS, elevated MDA levels, and significant reduction in the antioxidant enzymes SOD and CAT. These functional markers are sensitive to early damage and often precede evident morphological changes (Atamaniuk et al. 2013; Matviishyn et al. 2014; Liu et al. 2015; Miller and Zachary 2022). The imbalance between free radical production and antioxidant defenses can affect organelles, promote lipid peroxidation, and compromise cellular integrity even before histological damage is detectable by routine staining (Ohkawa et al. 1979; Shubin et al. 2016).

The imbalance between reactive species production and antioxidant activity leads to the formation of the hydroxyl radical (•OH), one of the most reactive and deleterious species, via the Fenton reaction. The simultaneous deficiency of SOD and CAT observed in this study impairs the cellular capacity to neutralize intermediate products generated by oxidative metabolism, intensifying lipid peroxidation and promoting protein and DNA cleavage (Yu and Anderson 1997; Chen et al. 2019).

In the kidneys, the toxic effects were more evident upon histomorphological evaluation. The treated group presented an accumulation of brownish granular pigment restricted to the proximal tubules and tubular necrosis, while alterations in the control group were of lesser intensity or absent. Based on morphology and localization, this pigment may correspond to either hemosiderin, a product of hemoglobin degradation and intracellular iron accumulation, or lipofuscin, which results from peroxidized membrane degradation under chronic cellular stress. Although 2,4-D is known to induce oxidative stress and renal injury, there are no specific reports in the literature linking this herbicide to pigment accumulation of this nature. Therefore, a direct causal relationship between pigment deposition and 2,4-D exposure cannot be definitively established (Brunk and Terman 2002; Miller and Zachary 2022; Jawad and Faraj 2022; Alqurashy et al. 2025).

The presence of tubular necrosis after short-term exposure reinforces the potential of 2,4-D to cause irreversible damage, even at levels considered safe (Shafeeq and Mahboob 2021). Despite this, renal stereology did not reveal differences in kidney volume or glomerular count, which is consistent with the multifocal and incipient nature of the observed lesions. This dissociation between functional and morphometric changes is common in subclinical exposures and has been described in similar models (Gallagher and Di Giulio 1991; Howard and Reed 2005; Atamaniuk et al. 2013; Kirici et al. 2022). Similar patterns have also been reported in fish exposed to 2,4-D, where biochemical and histological changes occur without corresponding stereological alterations (Menezes et al. 2015; Martins et al. 2024).

Collectively, these findings highlight the importance of validating subclinical and multifactorial biomarkers in reptiles exposed to agrochemicals, particularly in light of their slow biotransformation rates and heightened vulnerability in intensively managed agricultural environments. The inherently slow metabolism and low glomerular filtration rate characteristic of reptiles favor xenobiotic accumulation and intensify local toxic effects. This ectothermic physiology, combined with the prolonged elimination half-life of many contaminants, increases the risk of persistent toxic action even at low exposure levels. Moreover, regenerative processes that differ markedly from those observed in mammals render reptiles more susceptible to long-term cellular alterations resulting from acute or subchronic exposures. Chronic redox imbalance may compromise hepatic and renal function, affecting detoxification capacity as well as energy and water–salt homeostasis (Rheubert et al. 2011; Moeller et al. 2013; Dakhakhni et al. 2016; Holz 2020; Fasola et al. 2022; Caetano and Colli 2021).

Exposure to 2,4-D elicited distinct organ-specific responses, with the liver showing greater biochemical sensitivity and the kidney exhibiting greater histomorphological vulnerability. This pattern reflects physiological differences in xenobiotic biotransformation and excretion. Comparative studies involving rodents and fish report similar trends, identifying the liver as the primary target organ and the kidney as susceptible to segmental lesions associated with clearance processes (Troudi et al. 2011, 2012; Liu et al. 2015; Martins et al. 2024).

As a representative reptile species of the Cerrado biome, *T. torquatus* plays an important ecological role in regulating populations of insects and other small invertebrates—particularly ants, termites, and bees—thus contributing to biological control and trophic balance in local ecosystems (Teixeira and Giovanelli 1999; Carvalho et al. 2007; Gomides et al. 2013; Siqueira et al. 2013; Drago et al. 2020). Reptiles and amphibians more broadly function as key arthropod consumers, and their population declines may result in substantial community-level impacts (Cortés-Gomez et al. 2015). The physiological impairments observed following 2,4-D exposure suggest that reductions in lizard populations could generate cascading ecological effects, altering predator–prey dynamics and compromising regional biodiversity.

Species such as *T. torquatus* therefore function as sensitive bioindicators of environmental contamination, signaling ecological imbalance associated with indiscriminate agrochemical use. Evidence indicates that lizards can accumulate contaminants in their tissues and exhibit cellular damage markers in response to environmental pollution, reinforcing their value as ecotoxicological sentinels. Moreover, reptiles can reflect local contamination by metals and pesticides at the habitat level (Lambert 2005; Simbula et al. 2021). Understanding the sublethal effects of 2,4-D in reptiles thus contributes to a more comprehensive environmental risk assessment of this herbicide and underscores the need for sustainable agricultural practices and continuous wildlife monitoring. Declines in the abundance of *T. torquatus* and similar species may compromise essential ecological processes such as biological pest control and nutrient cycling. Herpetofauna, including reptiles and amphibians, contribute significantly to the regulation of pest insect populations in agroecosystems, acting as natural regulators(Ghosh et al. 2024). Some species also participate in seed dispersal, thereby supporting nutrient cycling and vegetation regeneration in Neotropical ecosystems (Valido and Olesen^ 2019^; Sazima and Sazima 2025). Agrochemical exposure has been linked to the loss of herpetofaunal species that provide these ecosystem services, emphasizing the importance of integrated ecotoxicological studies that address indirect ecological impacts and support sustainable agricultural practices (Wanger et al. 2023).

These findings reinforce the necessity of including ectothermic species in ecotoxicological monitoring programs, avoiding the simple extrapolation of toxicological data derived from mammals. *Tropidurus torquatus* proved to be a sensitive and suitable bioindicator model for assessing the environmental impacts of herbicides. Future studies should incorporate prolonged exposure scenarios, special histochemical stains, cellular biomarkers, and integrated functional assessments to elucidate mechanisms of tissue damage and recovery in native Neotropical herpetofauna. Beyond their ecotoxicological relevance, these results may contribute to the reassessment of regulatory pesticide limits from the perspective of tropical biodiversity. Given the widespread distribution of terrestrial reptiles in agricultural landscapes, their inclusion as official bioindicators in environmental monitoring protocols is both justified and urgent.

In summary, subchronic exposure to 2,4-D at a dose equivalent to the acceptable daily intake established for mammals induced redox imbalance and focal renal tubular injury in *Tropidurus torquatus*, characterizing an early pattern of functional toxicity in this ectothermic species. Although overt stereological alterations were not detected, the biochemical and histomorphological findings indicate that mammalian-based safety thresholds may not provide adequate protection for reptiles with distinct metabolic and excretory physiology. The nature of the tubular pigment observed in exposed animals could not be determined in the present study; therefore, a direct causal relationship with 2,4-D cannot be established, although its increased frequency warrants further investigation. These results reinforce the need to incorporate ectothermic species into ecotoxicological risk assessment and regulatory frameworks.

## Data Availability

The datasets generated and analysed during the current study are available from the corresponding author on reasonable request.
